# Clinical analysis of deceased donor liver transplantation in the treatment of hepatocellular carcinoma with segmental portal vein tumor thrombus: A long-term real-world study

**DOI:** 10.3389/fonc.2022.971532

**Published:** 2022-09-20

**Authors:** Meng Sha, Chen Chen, Chuan Shen, Seogsong Jeong, Han-yong Sun, Ning Xu, Hua-lian Hang, Jie Cao, Ying Tong

**Affiliations:** ^1^ Department of Liver Surgery, Renji Hospital, School of Medicine, Shanghai Jiao Tong University, Shanghai, China; ^2^ Department of Biomedical Informatics, CHA University School of Medicine, CHA University, Seongnam, South Korea; ^3^ Institute of Basic Medical Sciences, School of Medicine, CHA University, Seongnam, South Korea; ^4^ Institute for Biomedical Informatics, School of Medicine, CHA University, Seongnam, South Korea

**Keywords:** Hepatocellular carcinoma, portal vein tumor thrombus, deceased donor liver transplantation, segmental PVTT, lobar PVTT, microvascular invasion

## Abstract

**Background:**

Hepatocellular carcinoma (HCC) patients with portal vein tumor thrombus (PVTT) have conventionally been regarded as a contraindication for liver transplantation (LT). However, the outcomes of deceased donor liver transplantation (DDLT) in patients with segmental PVTT remain unknown. The aim of this study is to evaluate the feasibility and effectiveness of DDLT in the treatment of HCC with segmental PVTT.

**Methods:**

We retrospectively analyzed 254 patients who underwent DDLT for HCC in our institution from January 2015 to November 2019. To assess the risks of PVTT, various clinicopathological variables were evaluated. Overall (OS) and recurrence-free survival (RFS) analyses based on different PVTT types were performed in HCC patients.

**Results:**

Of the 254 patients, a total of 46 patients had PVTT, of whom 35 had lobar PVTT and 11 had segmental PVTT in second-order branches or below. Alpha-fetoprotein (AFP) level, tumor maximal diameter, histological grade, micro-vascular invasion (MVI), RFS, and OS were significantly different between the control and PVTT groups. Lobar PVTT was associated with unfavorable 5-year RFS and OS compared with MVI group (28.6% and 17.1%, respectively). Instead, no significant difference was observed between the segmental PVTT and MVI group in terms of 5-year RFS and OS (RFS: 36.4% vs. 40.4%, *p*=0.667; OS: 54.5% vs. 45.1%, *p*=0.395). Further subgroup analysis showed segmental PVTT with AFP levels ≤100 ng/ml presented significantly favorable RFS and OS rates than those with AFP level >100 ng/ml (*p*=0.050 and 0.035, respectively).

**Conclusions:**

In summary, lobar PVTT remains a contraindication to DDLT. HCC patients with segmental PVTT and AFP level ≤100 ng/ml may be acceptable candidates for DDLT.

## Introduction

Hepatocellular carcinoma (HCC) is the most common primary liver cancer, accounting for the third-highest cancer-associated deaths worldwide (1, 2). HCC invading into portal vein or branches is defined as portal vein tumor thrombus (PVTT), which has been reported in 44-62.2% of patients with HCC (3, 4). The median survival of untreated patients with PVTT is usually less than 6 months (5). Therefore, PVTT is considered an unfavorable prognostic factor for HCC (6, 7).

Liver transplantation (LT) is the potentially curative treatment for HCC, as it has the advantage of removing not only the tumor but also the cirrhotic liver (8). To achieve optimal results, a strict selection of patients, such as Milan criteria and UCSF criteria, has been established (9, 10). Among the criteria, the selection variables mainly focus on tumor size and tumor number, which exclude approximately 50% of patients with advanced HCC (11). Thus, expanded selection criteria of LT for HCC patients are expected.

Macro-vascular invasion like PVTT has previously been considered a contraindication for LT due to the high incidence of recurrence and poor prognosis following LT (12, 13). However, PVTT can be divided into different types including the main trunk of the portal vein, left or right portal vein, and segmental branches of the portal vein (14). Previous literature mainly focused on PVTT in the main trunk of the portal vein (15), and the outcomes of LT in HCC patients with segmental PVTT remained unknown. In addition, several recent studies reported survival benefits of living donor liver transplantation (LDLT) in HCC patients with PVTT (16, 17). However, the outcomes of these patients after deceased donor liver transplantation (DDLT) remained unclear. Therefore, we conducted the present study to evaluate the feasibility and effectiveness of DDLT for HCC patients with segmental PVTT and explored the survival outcomes.

## Materials and methods

### Study patients

**Figure 1:**
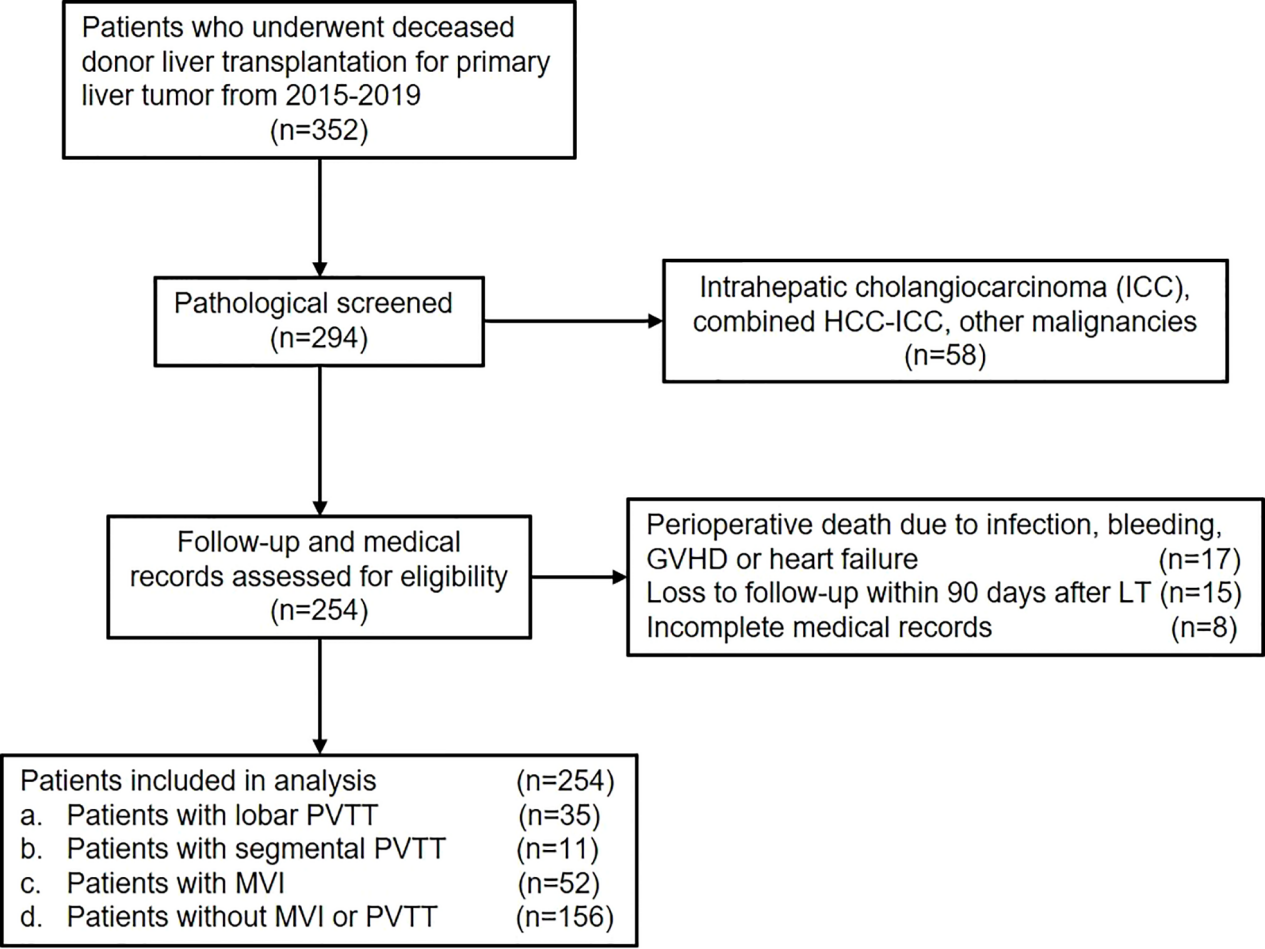
Study flow diagram.

Data of patients who underwent DDLT for HCC from January 2015 to December 2019 in Renji Hospital, School of Medicine, Shanghai Jiao Tong University (Shanghai, CN) were retrospectively reviewed. Adult patients diagnosed as HCC with or without PVTT who underwent DDLT were included in the present study (**Figure 1**). The exclusion criteria were as follows: (1) pathological diagnosis of intrahepatic cholangiocarcinoma (ICC), combined HCC-ICC or other malignancies; (2) perioperative death due to infection, bleeding, organ failure, etc.; (3) loss of follow-up within 90 days after LT; (4) incomplete medical records. Finally, patients were divided into different groups according to PVTT status. This study was conducted in accordance with the Declaration of Helsinki and was approved by the institutional ethics committee of Renji Hospital, School of Medicine, Shanghai Jiao Tong University.

### Diagnosis of PVTT

**Figure 2:**
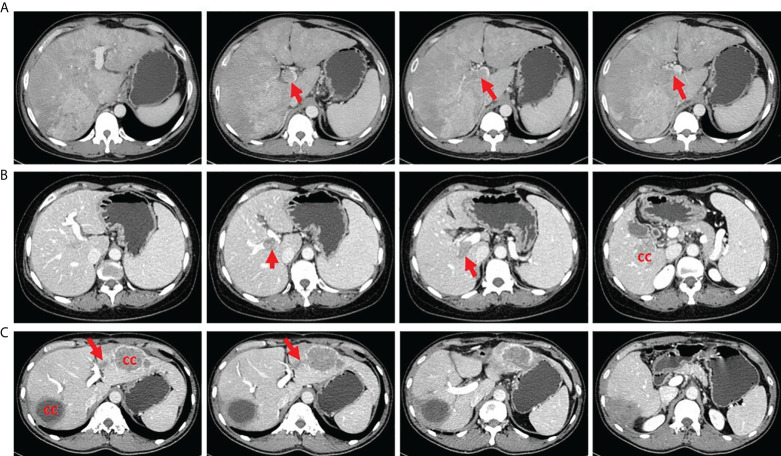
Tumor thrombus in different portal vein location. **(A)** A patient with tumor thrombus in the main trunk of portal vein. **(B)** A patient with tumor thrombus in the right portal vein. **(C)** A patient with tumor thrombus in the second branch of portal vein. CC, cancer center.

All patients with HCC who were scheduled for LT were evaluated preoperatively by blood tests, ultrasound, CT scan of the abdomen and chest, positron emission tomography (PET)-CT, and endoscopy to exclude extrahepatic lesions and distant metastasis. The diagnosis of PVTT was based on preoperative imaging examination and postoperative pathological confirmation. PVTT was classified into two types in our study: (1) a PVTT in the second-order branches of the portal vein or below was defined as segmental; (2) a PVTT in the right or left portal vein was defined as lobar. Patients with tumor thrombus in the main trunk of the portal vein or superior mesenteric vein were excluded (**Figure 2**). Patients with lobar PVTT proceeded to LT only with the preoperative consent of recipient after fully inform of disease status and possibility of recurrence.

### Data collection and follow-up

Preoperative baseline data and serological examinations including age, gender, hepatitis B virus, AFP level, carcinoembryonic antigen 19-9 (CA19-9) level and pretransplant loco-regional therapy were collected. Data of cirrhosis, tumor number, satellite lesions, tumor maximal diameter, histological grade and MVI were based on postoperative pathology.

An interleukin 2 (IL-2) receptor blocker was administered on the day of the operation and the fourth postoperative day. Postoperative immunosuppressive treatment included a regimen consist of a calcineurin inhibitor (cyclosporine or tacrolimus), mycophenolate mofetil (MMF) and steroids. Steroids were withdrawn 1 month after surgery, and MMF was withdrawn 3 months after surgery. Sirolimus was used 3 months after LT combined with a low level of calcineurin inhibitor.

All patients were followed up using liver function, serum AFP level as well as ultrasound monthly during the first year and every 3 months thereafter. To allow early detection of recurrence, CT or MRI scan of the chest and abdomen were performed once every 6 months. When tumor recurrence was suspected, PET-CT was conducted. Adjuvant therapy including transarterial chemoembolization (TACE), radiofrequency ablation, sorafenib or lenvatinib were permitted once tumor recurrence was confirmed. The main endpoint of this study was recurrence of tumor and death of patients. Data of overall (OS) and recurrence-free survival (RFS) were collected for all included patients.

### Statistical analysis

Continuous variables are presented as median with range and categorical variables are expressed as numbers with ratio. The correlations between PVTT category and clinicopathological characteristics were compared using χ2 or Fisher exact test for categorical variables and Student’s t-test or Mann-Whitney U test for continuous variables. Survival curves were estimated using the Kaplan–Meier analysis and compared with the log-rank test. To identify independent risk factors associated with recurrence-free survival and overall survival, Cox hazards proportional regression was performed. All statistical analyses were performed using SPSS (version 24.0). A *p*-value of <0.05 was considered statistically significant.

## Results

### Baseline characteristics

As shown in the flow chart, 352 patients who underwent DDLT for HCC from January 2015 to December 2019 were screened for the study. 58 patients who were pathologically diagnosed as ICC, combined HCC-ICC, and other malignancies were excluded. Another 40 patients didn’t meet the inclusion criteria due to perioperative death, loss of follow-up, and incomplete medical records. Finally, a total of 254 patients were included in the present study.

The median age of included patients was 51 years (22-75 years), and 223 (87.8%) were male. The majority of patients (228, 89.8%) had HBV infection and 7 (2.8%) had HCV infection. The median preoperative AFP and CA19-9 levels were 35.4ng/ml (0.7-60500ng/ml) and 24.9u/ml (0.6-2492u/ml), respectively. 58 patients (22.8%) underwent pretransplant loco-regional therapy to control or reduce tumor burden. Through postoperative pathology, most patients had underlying cirrhosis (222, 87.4%). Multiple tumors were present in 107 patients (42.1%) and 24 of them had satellite lesions (9.4%). The median maximal tumor diameters were 4cm (0.3-24cm) and 83 patients (32.7%) had poorly differentiated tumor grade. MVI was present in 78 patients (30.7%) and PVTT was confirmed in 46 patients (18.1%). Further, lobar and segmental PVTT were observed in 35 and 11 patients, respectively. Finally, patients were categorized into control group (no MVI or PVTT, n=156), MVI group (MVI only with no PVTT, n=52), lobar PVTT group (n=35), and segmental PVTT group (n=11).

Comparisons of clinicopathological variables based on recurrence status were first performed (**Table 1**). Significant differences were observed in preoperative AFP level (*p*<0.001), presence of cirrhosis (*p*=0.025), tumor number (*p*<0.001), satellite lesions (*p*<0.001), maximal diameter (*p*<0.001), histological grade (*p*<0.001) and presence of MVI (*p*<0.001) and PVTT (*p*<0.001).

**Table 1:** Characteristics of patients receiving LT with HCC by recurrence status.

Variable	Total ( n = 254 )	Non-recurrent ( n = 142 )	Recurrent ( n = 112 )	*p* value
Age, years	51 (22-75)	53 (22-75)	51 (29-75)	0.082
Gender, male, n (%)	223 (87.8)	125 (88.0)	98 (87.5)	0.898
HBV infection, n (%)	228 (89.8)	131 (92.3)	97 (86.6)	0.140
HCV infection, n (%)	7 (2.8)	3 (2.1)	4 (3.6)	0.703
AFP, ng/ml	35.4 (0.7-60500)	15.5 (0.7-55030)	119.4 (1.1-60500)	<0.001
CA19-9, u/ml	24.9 (0.6-2492)	21.9 (0.6-908.2)	28.4 (0.6-2492)	0.215
Cirrhosis, present, n (%)	222 (87.4)	130 (91.5)	92 (82.1)	0.025
Pretransplant treatment, present, n (%)	58 (22.8)	27 (19.0)	31 (27.7)	0.102
Tumor number, multiple, n (%)	107 (42.1)	45 (31.7)	62 (55.4)	<0.001
Satellite lesions, present, n (%)	24 (9.4)	4 (2.8)	20 (17.9)	<0.001
Maximal diameter, cm	4 (0.3-24)	3.5 (0.3-15)	6 (0.5-24)	<0.001
Histological grade, poor differentiated, n (%)	83 (32.7)	31 (21.8)	52 (46.4)	<0.001
MVI, present (%)	78 (30.7)	25 (17.6)	53 (47.3)	<0.001
PVTT				<0.001
No	208 (81.9)	132 (93.0)	76 (67.9)	
Segmental	35 (13.8)	6 (4.2)	29 (25.9)	
Lobar	11 (4.3)	4 (2.8)	7 (6.3)	

Data are median (range) unless indicated otherwise.

HBV, hepatitis B virus; HCV, hepatitis C virus; AFP, alpha-fetoprotein; CA19-9, carcinoembryonic antigen 19-9; MVI, micro-vascular invasion; PVTT, portal vein tumor thrombosis.

### Comparisons between MVI and PVTT

The clinicopathological characteristics were compared between the MVI and PVTT groups (**Table 2**). Preoperative AFP level did not differ between MVI and PVTT group (*p*=0.576). A significant difference in the maximal tumor diameter was observed between groups (control vs. MVI, *p*<0.001; MVI vs. PVTT, *p*=0.024; control vs. PVTT, *p*<0.001). Poorly differentiated tumor grade (control vs. MVI, *p*=0.001; control vs. PVTT, *p*<0.001) differed from those of the control group, but not between the MVI and PVTT groups (*p*=0.704). The presence of satellite lesions was significantly higher in the MVI group (control vs. MVI, *p*<0.001; MVI vs. PVTT, *p*=0.008).

**Table 2:** Comparisons of patients receiving LT with HCC by MVI and PVTT status.

Variable	Control ( n = 156 )	MVI ( n = 52 )	PVTT ( n = 46 )	*p* value control vs. MVI	*p* value MVI vs. PVTT	*p* value control vs. PVTT
Age, years	53 (29-74)	47 (22-75)	51 (34-66)	0.002	0.146	0.124
Gender, male, n (%)	133 (85.3)	50 (96.2)	40 (87.0)	0.036	0.197	0.773
HBV infection, n (%)	146 (93.6)	41 (78.8)	41 (89.1)	0.002	0.169	0.488
AFP, ng/ml	20.2 (1.3-60500)	75.7 (1.1-60500)	105.6 (0.7-60500)	0.006	0.576	0.002
CA19-9, u/ml	23.6 (0.6-2492)	35.9 (0.6-278)	22.5 (0.6-570.4)	0.254	0.305	0.981
Cirrhosis, present, n (%)	138 (88.5)	42 (80.8)	42 (91.3)	0.159	0.137	0.587
Pretransplant treatment, present, n (%)	37 (23.7)	10 (19.2)	11 (23.9)	0.503	0.573	0.978
Tumor number, multiple, n (%)	57 (36.5)	26 (50.0)	24 (52.2)	0.086	0.830	0.057
Satellite lesions, present, n (%)	7 (4.5)	14 (26.9)	3 (6.5)	<0.001	0.008	0.863
Maximal diameter, cm	3.5 (0.3-24)	4.8 (1-17)	7.5 (1-15)	<0.001	0.024	<0.001
Histological grade, poor differentiated, n (%)	36 (23.1)	24 (46.2)	23 (50.0)	0.001	0.704	<0.001
Recurrence, present, n (%)	45 (28.8)	31 (59.6)	36 (78.3)	<0.001	0.048	<0.001
Recurrence interval, months	53 (1-83)	26.5 (1-80)	9 (2-72)	<0.001	0.011	<0.001

Data are median (range) unless indicated otherwise.

HBV, hepatitis B virus; AFP, alpha-fetoprotein; CA19-9, carcinoembryonic antigen 19-9; MVI, micro-vascular invasion; PVTT, portal vein tumor thrombosis.

**Figure 3:**
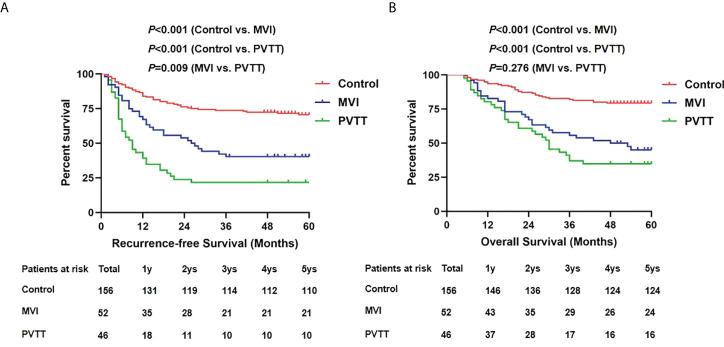
Recurrence-free survivals **(A)** and overall survivals **(B)** comparison among control, MVI and PVTT group.

We further analyzed the postoperative survival outcomes among groups as shown in **Figure 3**. During follow-up, tumor recurrence developed in 45 patients in the control group, 31 in the MVI group and 36 in the PVTT group. The 1-, 3- and 5-year RFS in the PVTT group was 39.1%, 21.7% and 21.7%, respectively, showing significant inferiority to the MVI group (67.3%, 40.4% and 40.4%, respectively, *p*=0.009) and control group (84.0%, 73.7% and 70.5%, respectively, *p*<0.001). Death was observed in 34 patients in the control group, 29 in the MVI group and 30 in the PVTT group. The OS was also poorer in the PVTT group than the control group (80.4%, 37%, 34.8% vs. 93.6%, 82.1%, 79.5%, *p*<0.001). However, no significant difference was observed in OS between PVTT group and MVI group (80.4%, 37%, 34.8% vs. 82.7%, 55.8%, 45.1%, *p*=0.276).

### Analysis of outcomes based on PVTT location

Next, we performed comparisons by the level at which the PVTT was located. As shown in **Table 3**, preoperative AFP (*p*=0.415), CA19-9 level (*p*=0.542) and pretransplant treatment (*p*=0.100) presented no difference between the lobar and segmental PVTT group. For tumor variables, neither tumor number (*p*=0.609) nor maximal diameter (0.703) differed between groups. The recurrence rate was a little higher in the lobar PVTT group (82.9% vs. 63.6%), but no statistical significance was observed (*p*=0.220). The recurrence interval was longer in the segmental PVTT group (*p*=0.049).

**Table 3:** Comparisons of patients receiving LT with HCC by different PVTT location.

Variable	Lobar ( n = 35 )	Segmental ( n = 11 )	*P* value
Age, years	51 (34-66)	50 (43-66)	0.648
Gender, male, n (%)	29 (82.9)	11 (100)	0.311
HBV infection, n (%)	31 (88.6)	10 (90.9)	1.000
AFP, ng/ml	105.7 (1.1-60500)	76.4 (0.7-60500)	0.415
CA19-9, u/ml	25.3 (0.6-570.4)	18.5 (1.6-173.7)	0.542
Cirrhosis, present, n (%)	31 (88.6)	11 (100)	0.559
Pretransplant treatment, present, n (%)	6 (17.1)	5 (45.5)	0.100
Tumor number, multiple, n (%)	19 (54.3)	5 (45.5)	0.609
Satellite lesions, present, n (%)	3 (8.6)	0 (0.0)	1.000
Maximal diameter, cm	8 2-15)	7 (1-12)	0.703
Histological grade, poor differentiated, n (%)	18 (51.4)	5 (45.5)	0.730
Recurrence, present, n (%)	29 (82.9)	7 (63.6)	0.220
Recurrence interval, months	8 (2-72)	20 (5-72)	0.049

Data are median (range) unless indicated otherwise.

HBV, hepatitis B virus; AFP, alpha-fetoprotein; CA19-9, carcinoembryonic antigen 19-9.

**Figure 4:**
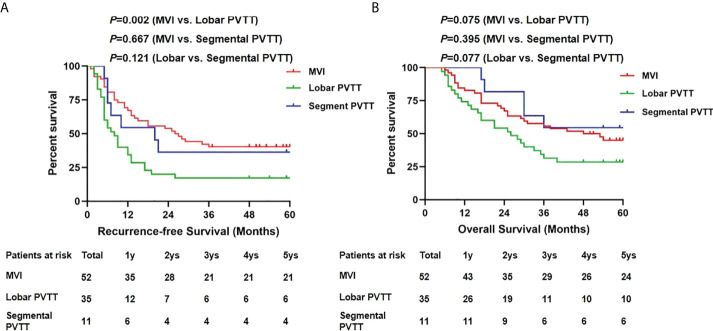
Recurrence-free survivals **(A)** and overall survivals **(B)** comparison among MVI, lobar and segmental PVTT group.

Next, we compared the RFS and OS by different PVTT status (**Figure 4**). No significant difference in 5-year RFS was detected between the segmental PVTT group and the MVI group (36.4% vs. 40.4%, *p*=0.667). However, the lobar PVTT group presented significantly worse RFS (17.1% vs. 40.4%, *p*=0.002) compared with the MVI group. For OS, the segmental PVTT group showed somewhat better outcomes than both the MVI group and the lobar PVTT groups, though no statistical significance was attained (*p*=0.395 and 0.077, respectively). The 1-, 3-, 5-year RFS and OS in the segmental PVTT group were 54.5%, 36.4%, 36.4% and 100%, 54.5%, 54.5%, respectively.

Since MVI was included in the PVTT group in our study, we further compared the results between groups of segmental PVTT, lobar PVTT and MVI. No significant difference of RFS (*p*=0.733) and OS (*p*=0.482) was observed between segmental group and segmental + MVI group (**Supplementary Figure 1**). Compared with segmental + lobar + MVI group, patients with lobar PVTT showed a worse RFS (*p*=0.027); while for OS, the results showed no significant difference (*p*=0.140) (**Supplementary Figure 2**).

### Risk of segmental PVTT with RFS and OS

We further investigated association of MVI and PVTT with recurrence-free survival and overall survival as shown in **Table 4**. Compared to MVI group, segmental PVTT group (HR, 1.195, 95%CI, 0.525-2.717) showed no significantly higher risk of HCC recurrence in the Cox hazards proportional regression. After adjustments for clinicopathological variables, the primary findings remained consistent (aHR, 1.974, 95%CI, 0.728-5.355). For overall survival, the segmental PVTT group presented no higher risk than MVI group (aHR, 0.996, 95%CI, 0.328-3.025) in the final adjustment model as well.

**Table 4:** Association of MVI and segmental PVTT with recurrence-free survival and overall survival among patients with HCC who underwent DDLT.

	MVI	Segmental PVTT	*p* value
Recurrence-free survival			
Event (%)	31 (59.6%)	7 (63.6%)	
HR (95% CI)	1.00 (reference)	1.195 (0.525, 2.717)	0.671
aHR (95% CI)^a^	1.00 (reference)	1.974 (0.728, 5.355)	0.182
Overall survival			
Event (%)	29 (55.8%)	5 (45.5%)	
HR (95% CI)	1.00 (reference)	0.666 (0.257, 1.728)	0.403
aHR (95% CI)^a^	1.00 (reference)	0.996 (0.328, 3.025)	0.995

HR calculated using Cox hazards proportional regression.

a Calculated after adjustments for age, sex, hepatitis B virus infection, alpha-fetoprotein, carcinoembryonic antigen 19-9, liver cirrhosis, pretransplant treatment, tumor size and number, satellite lesion, and histological grade.

MVI, micro-vascular invasion; PVTT, portal vein tumor thrombosis; HR, hazard ratio; CI, confidence interval; aHR, adjusted hazard ratio.

### Univariate and multivariate cox regression analyses of RFS and OS

To investigate the independent risk factors for RFS and OS, univariate and multivariate analyses based on Cox regression were conducted as shown in **Tables 5** and **6**. For RFS, univariate analysis showed that significant prognostic factors included preoperative AFP level, cirrhosis, tumor number, satellite lesions, maximal diameter, histological grade and PVTT. Through multivariate analysis, preoperative AFP level>100ng/ml (*p*=0.004), absence of cirrhosis (*p*<0.001), satellite lesions (*p*=0.004), maximal diameter>5cm (*p*=0.010) and presence of PVTT (*p*<0.001) were identified as independent risk factors for RFS. Similarly, the above five factors included preoperative AFP level>100ng/ml (*p*<0.001), absence of cirrhosis (*p*=0.002), satellite lesions (*p*=0.027), maximal diameter>5cm (*p*=0.037) and presence of PVTT (*p*=0.002) were also determined as independent risk factors for OS.

**Table 5:** Univariate and multivariate analysis of prognosis factors for recurrence-free survival in patients who underwent deceased LT.

Variables	Univariate analysis			Multivariate analysis		
	HR	95%CI	P value	HR	95%CI	P value
Age (≤50 vs. >50)	0.748	0.517-1.084	0.126			
Gender (Male vs. Female)	1.024	0.585-1.792	0.935			
HBV infection (Absence vs. Presence)	0.690	0.400-1.188	0.181			
Preoperative AFP level (ng/ml) (≤100 vs. >100)	2.539	1.748-3.687	**<0.001**	1.803	1.202-2.706	**0.004**
Preoperative CA19-9 (U/ml) (≤20 vs. >20)	1.304	0.892-1.905	0.171			
Cirrhosis (Absence vs. Presence)	0.496	0.306-0.806	**0.005**	0.393	0.235-0.656	**<0.001**
Pretransplant treatment (Absence vs. Presence)	1.314	0.869-1.989	0.196			
Tumor number (Single vs. Multiple)	2.104	1.448-3.056	**<0.001**	1.530	1.000-2.342	0.050
Satellite lesions (Absence vs. Presence)	3.352	2.058-5.460	**<0.001**	2.351	1.320-4.185	**0.004**
Maximal diameter (cm) (≤5 vs. >5)	2.947	2.030-4.279	**<0.001**	1.732	1.139-2.632	**0.010**
Histological grade (well and moderate vs. poor)	2.395	1.650-3.476	**<0.001**	1.368	0.904-2.069	0.138
PVTT (Without PVTT vs. Lobar and segmental PVTT)	3.564	2.383-5.332	**<0.001**	2.813	1.782-4.442	**<0.001**

A p-value of <0.05 is presented in bold values.

**Table 6:** Univariate and multivariate analysis of prognosis factors for overall survival in patients who underwent deceased LT.

Variables	Univariate analysis			Multivariate analysis		
	HR	95%CI	P value	HR	95%CI	P value
Age (≤50 vs. >50)	0.731	0.487-1.098	0.132			
Gender (Male vs. Female)	0.912	0.486-1.712	0.775			
HBV infection (Absence vs. Presence)	0.963	0.499-1.856	0.910			
Preoperative AFP level (ng/ml) (≤100 vs. >100)	3.043	2.013-4.602	**<0.001**	2.322	1.475-3.656	**<0.001**
Preoperative CA19-9 (U/ml) (≤20 vs. >20)	1.186	0.783-1.796	0.422			
Cirrhosis (Absence vs. Presence)	0.494	0.292-0.837	**0.009**	0.409	0.235-0.712	**0.002**
Pretransplant treatment (Absence vs. Presence)	1.010	0.626-1.630	0.967			
Tumor number (Single vs. Multiple)	1.869	1.243-2.809	**0.003**	1.393	0.873-2.223	0.164
Satellite lesions (Absence vs. Presence)	3.284	1.932-5.580	**<0.001**	2.046	1.083-3.868	**0.027**
Maximal diameter (cm) (≤5 vs. >5)	2.790	1.855-4.196	**<0.001**	1.645	1.031-2.623	**0.037**
Histological grade (well and moderate vs. poor)	2.361	1.570-3.551	**<0.001**	1.316	0.831-2.085	0.242
PVTT (Without PVTT vs. Lobar and segmental PVTT)	2.863	1.850-4.431	**<0.001**	2.145	1.312-3.506	**0.002**

A p-value of <0.05 is presented in bold values.

### Subgroup analysis of segmental PVTT

**Figure 5:**
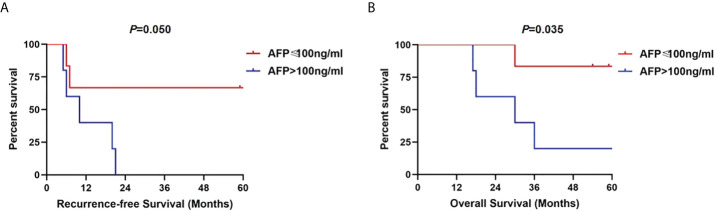
Recurrence-free survivals **(A)** and overall survivals **(B)** comparison in subgroup analysis of patients with segmental PVTT based on AFP level of 100 ng/ml.

**Figure 6:**
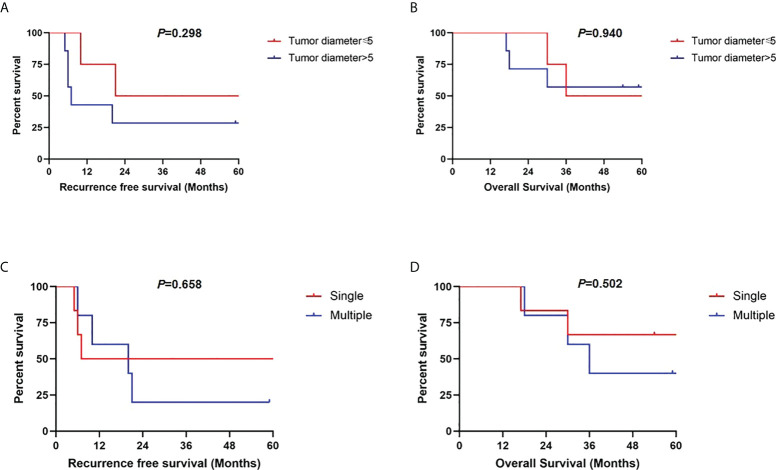
Recurrence-free survivals and overall survivals comparisons in subgroup analysis of patients with segmental PVTT based on maximal tumor diameter of 5 cm **(A, B)** and tumor numbers **(C, D)**.

Further, we conducted subgroup analysis in segmental PVTT group by different tumor characteristics. As shown in **Figure 5**, patients with AFP levels >100 ng/ml presented significantly worse RFS (*p*=0.050) and OS rates (*p*=0.035) than those with AFP level ≤100 ng/ml. However, no long-term survival differences were detected based on maximal tumor diameter of 5cm (RFS, *p*=0.298; OS, *p*=0.940). Similarly, no RFS and OS benefits were observed in groups of single tumor lesion (RFS, *p*=0.658; OS, *p*=0.502) compared with multiple tumor lesions (**Figure 6**).

## Discussion

Advanced HCC combined with PVTT has been shown in 44-62.2% of HCC patients (4). According to the Barcelona Clinic Liver Cancer guideline, sorafenib is regarded as the only treatment option (18, 19), while surgical treatment including LT is a contraindication due to the increased risk of spread of cancer cells into bloodstream, resulting in negative outcomes. With aim to prolong survivals of patients with PVTT through LT, several recent studies have expanded the LDLT indications in these patients and reported acceptable results (20–22). However, the outcomes based on different PVTT types have few been reported. In addition, whether HCC with PVTT can be expanded in DDLT remains unknown as well. Therefore, we conducted the present study to determine whether HCC with different PVTT types is feasible for DDLT.

A total of 254 patients were included in the current study and divided into groups based on MVI and PVTT status. Tumor characteristics were first analyzed among groups. Unsurprisingly, patients in MVI and PVTT groups exhibited higher AFP level, larger tumor size and poorer histological grade than the control group. However, it is noted that these variables showed no difference between the MVI and PVTT groups. Generally, MVI is defined as cancer cells within vascular endothelium identified microscopically (23). Through the bloodstream, cancer cells expanded from micro-vessels into macro-vessels, then proceed to the branches of PV and later involve the first-order of PV and finally the main trunk (24). The flow of tumor cells rather than proliferation may explain the similarity of tumor characteristics between the MVI and PVTT groups. Further survival analysis showed that the RFS and OS rates of PVTT group were lower than that of MVI group and control group. The disappointing results seem consistent with previous studies of LT in HCC with PVTT (25, 26). Nevertheless, the level at which PVTT was located should not be ignored, which calls for more strict criteria and stratified study.

We further divided the patients based on segmental and lobar PVTT and compared the results. Preoperative AFP level, tumor numbers, maximal diameter and histological grade did not differ between groups. However, the lobar PVTT group presented significantly worse long-term outcomes compared with the MVI group, with the 5-year RFS and OS of only 17.1% and 28.6%. The unfavorable results may exclude lobar PVTT as indication for LT. On the contrary, no significant difference of RFS and OS were observed between the segmental PVTT group and MVI group, with somewhat better OS in patients with segmental PVTT. As known, tumor cells extending from micro-vessels to macro-vessels through blood stream is one of the major routes of tumor recurrence and metastasis. Therefore, earlier and easier dissemination and spread of tumor cells from macro-vessels of lobar portal vein may contribute to the unfavorable outcomes of these patients. While tumors restricted in segmental portal vein may share the similar biological activity as MVI, which explains the comparable outcomes between segmental PVTT and MVI group. Since MVI is not known before surgery and HCC patients are expected to have 5-year survival rate of at least 50% (27), patients with detected segmental PVTT presenting 5-year OS of 54.5% should be considered as potential candidates for LT.

Preoperative AFP level has been accepted as one of the tumor biological indicators to predict tumor recurrence and select patients for LT (28, 29). In the multivariate analyses of risk factors, preoperative AFP level >100ng/ml was identified one of the independent risk factors for both RFS and OS. Therefore, we further analyzed segmental PVTT group according to AFP cutoff of 100 ng/ml. It turns out that patients with AFP levels ≤100 ng/ml presented significantly favorable RFS and OS rates than those with AFP level >100 ng/ml. Surprisingly, neither tumor diameter nor tumor number serves as predictors to further select patients with segmental PVTT. The results may suggest that tumor biological characteristics of AFP level plays more important role than morphological variables in tumor recurrence and prognosis (30, 31). Therefore, HCC patients with segmental PVTT and AFP level ≤100 ng/ml are acceptable for selecting criteria of LT.

Our study has some potential limitations. Firstly, it is a retrospective study with an imbalance in the group population and a limited number of patients. Multicenter large-scale studies are needed to confirm the results. Secondly, detailed preoperative downstaging procedures including TACE or transarterial radioembolization (TARE) were unavailable in data collection, which may cause bias in results. In addition, PVTT below the second-order branches is difficult to be identified accurately in preoperative imaging. These patients require further exploration with more precise detection.

In conclusion, HCC patients with segmental PVTT may be acceptable candidates for DDLT. Low level of preoperative AFP level may provide better results in selecting patients with segmental PVTT. Future exploration in large-scale, prospective studies is required to develop more appropriate criteria of LT for HCC patients and provide a favorable prognosis.

## Data availability statement

The raw data supporting the conclusions of this article will be made available by the authors, without undue reservation.

## Ethics statement

The studies involving human participants were reviewed and approved by Renji Hospital, School of Medicine, Shanghai Jiao Tong University. The patients/participants provided their written informed consent to participate in this study.

## Author contributions

Conception and design: MS and JC. Administrative support: SJ. Provision of study materials or patients: H-YS, H-LH and NX. Collection and assembly of data: MS and CS. Data analysis and interpretation: MS, CC and YT. Manuscript writing: All authors. All authors contributed to the article and approved the submitted version.

## Funding 

This work was supported by the National Natural Science Foundation of China (81902379), Cultivation Foundation of Renji Hospital (RJPY-LX-011) and National Key Research on Precision Medicine of China (2018ZX10723204).

## Conflict of interest

The authors declare that the research was conducted in the absence of any commercial or financial relationships that could be construed as a potential conflict of interest.

## Publisher’s note

All claims expressed in this article are solely those of the authors and do not necessarily represent those of their affiliated organizations, or those of the publisher, the editors and the reviewers. Any product that may be evaluated in this article, or claim that may be made by its manufacturer, is not guaranteed or endorsed by the publisher.
