# Supplementary figures and images for: Clinical analysis of deceased donor liver transplantation in the treatment of hepatocellular carcinoma with segmental portal vein tumor thrombus: A long-term real-world study
Source: Front Oncol. 2022 Sep 20;12:971532. doi: 10.3389/fonc.2022.971532 (PMC9530398; doi:10.3389/fonc.2022.971532)

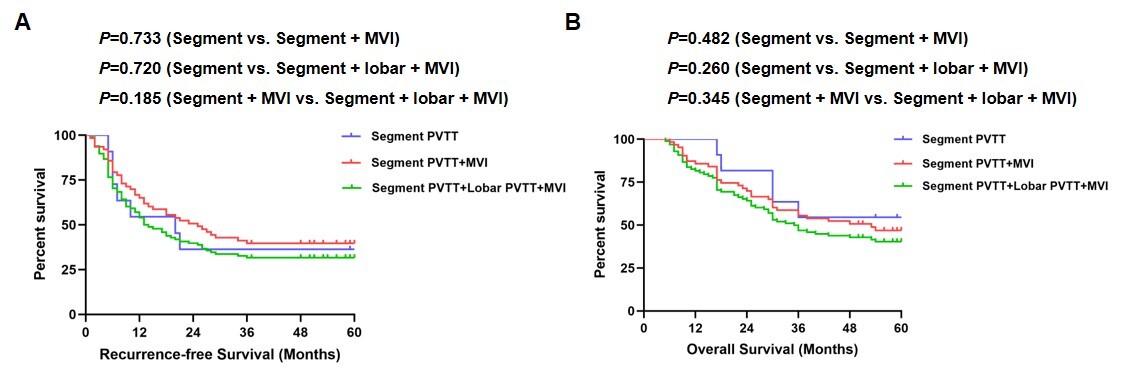

Supplement: Supplementary file 1 [file Image_1.jpeg]

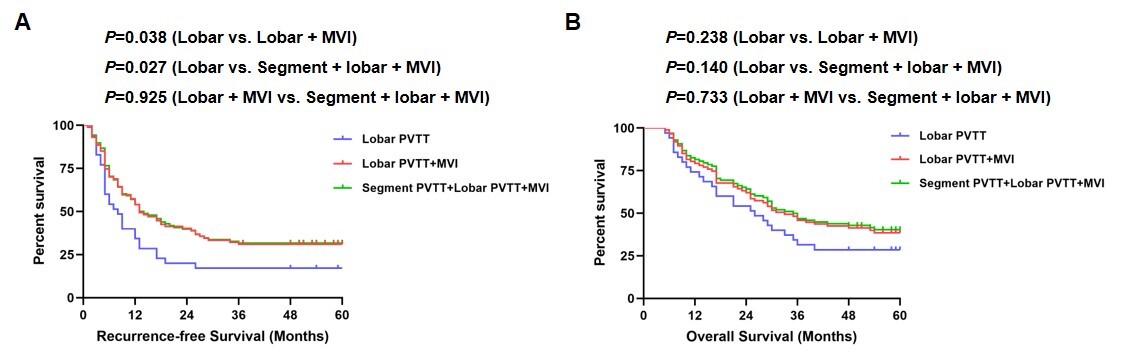

Supplement: Supplementary file 2 [file Image_2.jpeg]
